# From development to taxonomy: the case of *Sciaenacotyle pancerii* (Monogenea: Microcotylidae) in the Mediterranean meagre

**DOI:** 10.1017/S0031182022000865

**Published:** 2022-11

**Authors:** Mar Villar-Torres, Francisco Esteban Montero, Paolo Merella, Giovanni Garippa, Santino Cherchi, Juan Antonio Raga, Aigües Repullés-Albelda

**Affiliations:** 1 Cavanilles Institute of Biodiversity and Evolutionary Biology, Science Park, University of Valencia, 46980 Paterna, Valencia, Spain; 2 Parassitologia e Malattie Parassitarie, Dipartimento di Medicina Veterinaria, Università di Sassari, via Vienna 2, 07100 Sassari, Italy

**Keywords:** Aquaculture, clamp pair number, haptoral asymmetry, morphological variability, Polyopisthocotylea, Sciaenidae, species identification

## Abstract

The microcotylid *Sciaenacotyle pancerii* is a pathogenic monogenean infecting *Argyrosomus regius*, a candidate for species diversification in the Mediterranean aquaculture. Life-history stages of *S. pancerii* commonly co-occur in field infections, but to date, morphological data have only been provided for oncomiracidia and adults although identifying life-history stages can be useful in infection management. A total of 114 specimens of *S. pancerii* were analysed to characterize the developmental events and to assess morphological and morphometric variations before and after maturity. The post-larval development of *S. pancerii* is characterized by: expansion and bifurcation of the gut, loss of the larval haptor, protandrous development of the genitalia and vitellaria formation. The size variability of larval hooks, hamuli and germanium of *S. pancerii* is firstly reported and dimensional ranges of parasite body, haptor, testes, posteriormost clamps and eggs are widened. The size of most of the diagnostic features of *S. pancerii* significantly increases after parasite maturity and therefore, only those specimens with more than 116 clamps should be considered for minimising development-related variability in size. The high number of clamps, their fast development and the asymmetry in their size and arrangement suggest that *S. pancerii* may use a mixed attachment strategy between the closely related microcotylids and heteraxinids. This combination of features may be host related and linked to the gill structure of the sciaenid fish and the phylogenetic position of the genus *Sciaenacotyle*; distant from other microcotylids while close to heteraxinid species.

## Graphical abstract

**Figure:**
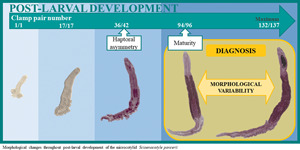

## Introduction

Monogeneans stand out as important threats in fish farms, where infections caused by pathological agents are recurrent (Nowak, 2007). Among monogenean parasites, microcotylids are considered particularly hazardous in marine aquaculture because of their impacts on fish health and growth (Ternengo and Katharios, 2008; Ternengo *et al*., 2010; Ogawa, 2015; Shinn *et al*., 2015). In the Mediterranean, the microcotylid *Sciaenacotyle pancerii* (Sonsino, 1891) was originally reported in shi drum [*Umbrina cirrosa* (L.)] (see Sonsino, 1891; Parona, 1912; Palombi, 1949) and subsequently found in wild meagre [*Argyrosomus regius* (Asso, 1801)] (see Ktari, 1970). Recent studies also report massive infections of *S. pancerii* in farmed meagre (Merella *et al*., 2009; Ternengo *et al*., 2010), an emerging species in Mediterranean aquaculture (Rigos and Katharios, 2010). Therefore, related studies deal with parasite effects in farmed hosts (Merella *et al*., 2009; Ternengo *et al*., 2010) and usually disregard morphological analysis, despite its relevance for diagnosis and infection management.

In the field, all life-history stages of *S. pancerii* (i.e. adults, juveniles and post-larvae) occur in mixed infections, but specific identification relies on the morphological analysis of adult specimens (Sonsino, 1891; Ktari, 1970). Understanding sources of morphological variability, such as the development in monogeneans, may help determine consistent diagnostic features (Thoney, 1986) and can be critical for decision making in aquaculture. Previous morphological analyses on *S. pancerii* include the descriptions of the oncomiracidium, the adult and a post-larval stage with 12 pairs of clamps (Ktari, 1970), but the development of this species or any other microcotylid with asymmetrical haptor is still unexplored. Due to the relevance of the haptor morphology in parasite attachment (Kearn, 2004), further developmental studies are timely and required. Therefore, this study aims at understanding the attachment strategy and improving the morphological description of *S. pancerii* by describing the main morphological changes occurring during the post-larval development and comparing the development of symmetrical and asymmetrical microcotylids.

## Materials and methods

Specimens of the current study were provided and identified by the authors of a previous study (Merella *et al*., 2009). *Sciaenacotyle pancerii* individuals were obtained from 36 heavily infected meagre (*A*. *regius*) collected during an epizootic episode (mean intensity above 100 parasites/fish) in a fish farm off the north-east of Sardinia (41°00′N, 8°52′E to 39°59′N, 9°41′E) between September and October 2007. Based on the quality of the specimens and their developmental stage, a total of 114 ethanol-fixed parasites were selected for morphological analysis. Worms were examined on permanent or temporary mounts, according to their developmental degree. Most parasites (*N* = 94, > 6 pairs of clamps) were stained with iron acetocarmine, dehydrated through a graded alcohol series, cleared in dimethyl phthalate and mounted in Canada balsam. Additionally, some early post-larval stages (*N* = 20, ⩽ 28 pairs of clamps) were mounted unstained in Kaiser's glycerol-gelatine (Sigma-Aldrich, USA). Morphological analysis of post-larvae specimens of *S. pancerii* was performed using a Leica DMR light microscope (Wetzlar, Germany; 100–1000×) and data on the main developmental events were recorded. Morphological changes in key structures for attachment (oral suckers, larval haptor and clamps), feeding (pharynx, oesophagus, gut and caeca) and reproduction (genital atrium, vitellaria, uterus, vagina, germarium, testes and intrauterine eggs) were registered. The clamp pair number of each parasite and measurements of the main structures were obtained from drawings using ImageJ 1.48v software (Rasband, 1997–2016). Following previous studies on microcotylids, the clamp pair number ‘CPN’ was used as an estimator of the age of the specimens. Parasites will be hereafter referred to according to their developmental degree as ‘CPN#’ in symmetrical stages or ‘CPN#short side/#long side’ in asymmetrical stages, being ‘#’ the number of clamps in each haptor side. The correlation between body length (mm) and the CPN during development was analysed. To account for post-maturity variability, bivariate relationships between the CPN and body size, haptor length and the number or size of the morphological structures in mature stages of *S. pancerii* were also analysed with the statistical significance set at *P* < 0.05. For statistical purposes, the number of clamps at the longer side of the haptor side is used as CPN. Kendall's correlation coefficient was calculated in R v.3.1.2 software (R Development Core Team, 2014). The size of the morphological structures is expressed as length × width in micrometres unless otherwise stated.

## Results

One hundred fourteen parasites were morphologically analysed to describe the main developmental events in the post-larval development of *S. pancerii*. Most of the parasites were immature stages (from CPN 1 to CPN 91; *N* = 68), while 40% of the specimens were mature stages (from CPN 94/96 to CPN 132/137; *N* = 46).

**Fig. 1.**
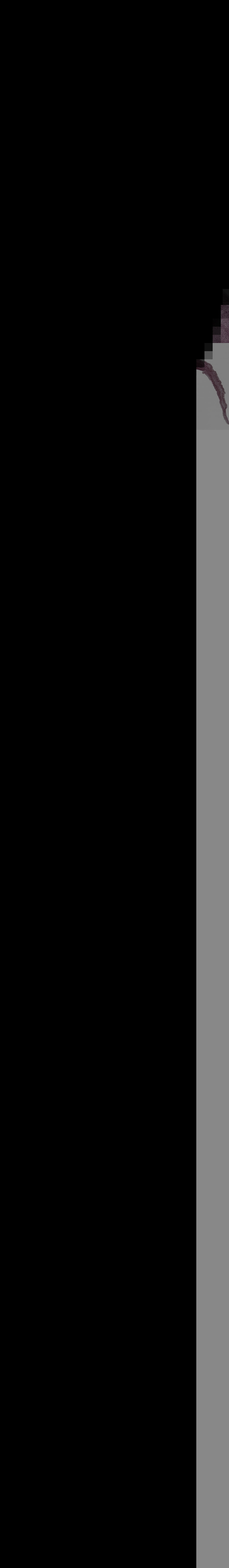
Morphology of different post-larval stages of *Sciaenacotyle pancerii.* (A) Earliest clamp-bearing stage (CPN1). (B) CPN 13 with the terminal lappet. (C–D) CPN 36 with a detail of the genital atrium, (E–F) CPN 50 with a detail of the testes. (G–H) CPN 60 with a detail of the uterus and germarium. (I) Mature specimen without eggs. (J) Mature specimen with intrauterine eggs. Scale bars = (A, D) 50 *μ*m, (F, H) 100 *μ*m, (B) 200 *μ*m, (C, E, G) 500 *μ*m, (I) 1000 *μ*m, (J) 2000 *μ*m.

The youngest specimen (CPN 1) is 275 long × 50 wide. The first pair of clamps (42 × 52) develop anterior to the posterior hooks (35 long) and larval hamuli (34 × 21) (Fig. 1A). The CPN 1 specimen shows the postero-lateral hooklets in the posterior-most region of the haptor whereas lateral hooklets are not observed. As parasite grew, new clamps develop towards the anterior end of the haptor, where between 1 and 5 developing pairs of clamps can be observed: 1–2 up to CPN 21; 2–4 up to 50; 2–5 up to CPN 111; 2–4 up to CPN 114/116; 1–2 up to CPN 132/137. The terminal lappet with posterior hooks and hamuli falls between CPN 10 and CPN 17 stages (*N* = 20; Table 1; Figure 1B). Posterior hooks (35–42 long) and larval hamuli (33–42 × 21–25) barely grow through post-larval development from CPN 1 to CPN 17 stages (*N* = 26). The posteriormost clamps are the smallest and the first to be developed and do not grow throughout development, overlapping in size from CPN 1 to CPN 132/137 stages (32–48 × 50–62). Germinal clamps develop at the anterior haptor end and increase gradually in size. The largest clamp pair is generally located between the anterior third and half of the haptor. Clamps anterior and posterior to the largest clamp pair are progressively smaller. The size of the anteriormost clamps is always larger than the posteriormost clamps. The size of the largest clamp increases throughout development from 39 × 58 at CPN 11 to 72 × 140 at CPN 109/112 parasites. Both sides of the haptor show a similar number and size of clamps until CPN 39 stages. A slight haptoral asymmetry in the number of clamps, either on the left or the right side, was detected in older parasites, especially in mature stages (Fig. 1I and J). The difference in the number of clamps between the long and the short haptor side ranges between 2 and 13 (CPN 63/61 and CPN 103/116, respectively). The clamps in the longer side of the haptor are also slightly wider (between +5 and +10 *μ*m width, overall), especially in the anterior half of the haptor. The specimen with the maximum number of clamps accounts for 132/137 clamps.

**Table 1.** Developmental stage and body length of the specimens at the occurrence of the main events in the post-larval development of *Sciaenacotyle pancerii.* Developmental stages designated by the clamp pair number (CPN)

	CPN	Parasite length (*μ*m)
Gut bifurcation	6–10	614–1097
Terminal lappet fall	10–17	690–1467
Genital atrium	31–49	1927–3804
Testes	36–51	2331–4495
Uterus	50–55/59	3434–4495
Germarium	58–84/90	3135–7406
Vitelline reservoir	58–94/96	3135–7406
Egg formation	94/96–109/115	6169–11 772

CPN in asymmetrical stages referred to as the number of clamps at the short/long haptor side.

A pair of oval-shaped buccal suckers (20 × 14), as well as a circular pharynx (13 × 13), are already visible at CPN 1 stages (Fig. 1A). The septum is differentiated in each sucker from CPN 11 stages. Biloculate suckers (77 × 52) are completely defined at CPN 36 stages and reach their maximum size (150 × 109) at CPN 111/119 specimens. The pharynx increases in size and changes from circular in CPN 1 stages to pear shaped in juveniles before maturity (CPN 81; 76 × 71). Maximum size is reached after maturity (CPN 123/128; 124 × 94). The gut is diffuse and extends to the haptor in the early post-larval stages (CPN 1 – CPN 6; *N* = 4). From CPN 10 stages, the bifurcated gut, as well as the oesophagus and caeca, are clearly distinguishable.

Regarding the reproductive structures, *S. pancerii* follows a protandrous development sequence (Table 1). The genital atrium and the vagina are the first structures developed and are distinguishable from < 35 CPN. The primordium of the genital atrium, unarmed and circular shaped, was first recognized at CPN 31 stages. By the CPN36 (Fig. 1C and D) the genital atrium, transversally elongated and constricted, was clearly defined (80 × 84) and the genital spines had developed. The genital atrium grows and reaches the definitive size (190 × 387) at CPN 124/134. The primordia of testes are also apparent at CPN36. Testes are completely defined (21 × 38; *N* = 38) with visible spermatids at CPN 51 (Fig. 1E and F). Testes increase in size and number through development to their maximum (79 × 110; *N* = 71) between CPN 122/131 and CPN 124/134 stages (*N* = 6). Female reproductive organs begin to develop between CPN 50 and CPN 59 stages (*N* = 4; Figure 1G and H) when the uterus is developed and the primordia of the germarium and vitelline reservoir were first observed. In course of development, the germarium grows from 682 long at CPN 84/90 to 1604 long at CPN 111/119 and vitelline glands extend. Completely mature parasites (Fig. 1I and J) were detected from CPN 94/96 when the first intrauterine eggs (*N* = 1) are observed. A maximum of 111 intrauterine eggs (mean per egg = 180 × 69; *N* = 23) were found in CPN 109/112 specimens.

**Fig. 2.**
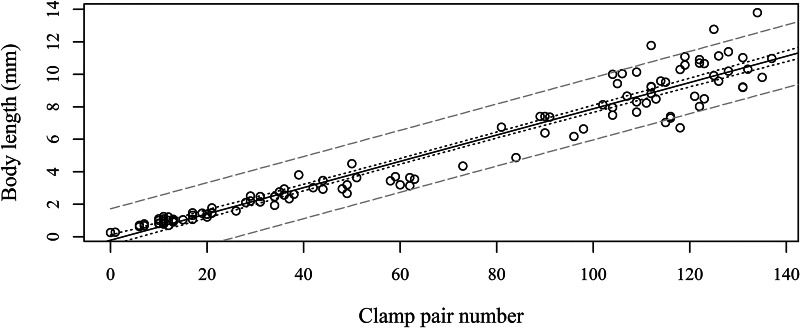
Body length of *Sciaenacotyle pancerii* as a function of the number of clamp pairs. The clamp-pair number in asymmetrical stages referred as the number of clamps at the longer haptor side. Dotted lines represent the 95% confidence interval. Dashed lines represent 95% prediction interval. Oncomiracidia length from Ktari (1970).

The clamp pair number strongly correlates with body length throughout parasite development (Fig. 2). The relationship between the 2 variables is described by a linear function (*R*^2^ = 0.939; *Y* = 0.0809*x* – 0.2077). Positive correlations between total body size (length and width) (*P* < 0.001), the largest clamp size (*P* < 0.05) and haptor length (*P* < 0.001) with the CPN were recorded throughout post-maturity development. The size of suckers (*P* < 0.05), pharynx (*P* < 0.05), genital atrium (*P* < 0.01), testes (*P* < 0.01) and germarium (*P* < 0.01) and the number of testes (*P* < 0.01) also increased significantly with the CPN after parasite maturity. No significant correlations were found between the rest of the morphological variables of *S. pancerii* nor the number of intrauterine eggs in mature stages and the CPN (*P* > 0.05).

## Discussion

Developmental changes in the morphology of *S. pancerii* are poorly known though they can be helpful for understanding parasite behaviour and effects and assessing the infection status. The post-larval development of *S. pancerii* is herein described for the first time. New records on *S. pancerii* provide novel developmental information on a genus with special morphological features among microcotylids (i.e. large body length, high clamp pair number and asymmetrical haptor) and thus, enhance the current knowledge on the former family.

The analysis of the post-larval development of *S. pancerii* provides for the first time the size variability of the larval hooks, hamuli and germarium during development (only available in figures to date; see Ktari, 1970), and updates the dimensions of the parasite body and attachment structures of adult specimens. The size of the main morphological features of adult stages of *S. pancerii* is generally consistent with the original taxonomic description (Sonsino, 1891) and the redescription (Ktari, 1970) of the species as well as with recent reports where it was identified (Merella *et al*., 2009). Posteriormost clamps are, however, smaller (shorter and narrower) than previously described (60 × 60 *μ*m; Ktari, 1970) and tend to be wider than long, as described for the rest of microcotylids (Mamaev, 1989). The size variability of most morphological structures of adult *S. pancerii* was higher than previously reported. To date, metrical data on *S. pancerii* is exclusively based on specimens infecting *U. cirrosa* (Sonsino, 1891; Ktari, 1970) while this is the first morphological analysis of this monogenean on *A. regius*, thus the higher variability can be related to the host species. Sample size, host size (Thoney, 1988) or environmental factors at the sampling locality (Brazenor and Hutson, 2015) may also contribute to this size variability. The new measurements of adult specimens of *S. pancerii* allow widening the morphological ranges of some taxonomic characters at the top and low margins and should be updated and considered for species identification (see Table 2). To date, 20 microcotylid species, belonging to the genera *Anchoromicrocotyle*, *Cynoscionicola*, *Diplostamenides*, *Microcotyloides*, *Pauciconfibula* and *Sciaenacotyle* have been described from sciaenid fish (see Gibson *et al*., 2005; WoRMS Editorial Board, 2022). The genus *Sciaenacotyle* differs from other microcotylids from sciaenid hosts mainly by the shape and armature of the genital atrium (Fujii, 1944; Price, 1962; Unnithan, 1971; Bravo-Hollis, 1981; Mamaev, 1989; Chisholm *et al*., 1991). The same feature allows for discriminating *Sciaenacotyle* spp. together with its hosts and geographic distribution (Hayward *et al*., 2007). Moreover, present findings on *S. pancerii* development reveal that some morphological features of adults, commonly used for species diagnosis within the Microcotylidae (e.g. the number of testes or clamps) (Mamaev, 1989) can be difficult to ascertain or be variable after maturity. Testes in *S. pancerii* have a poorly defined contour, are closely overlapped and are partially covered by the vitelline fields thus hindering the counting. The number of testes increases from 38 in young to 71 in old adults, which may also be confusing. Based on the size variations of *S. pancerii* throughout adult development, we recommend setting the number of clamps on the short side to a minimum of 116 to offer consistent and comparative morphological data.

**Table 2.** Measurements (in micrometres) of distinctive features of mature stages of *Sciaenacotyle pancerii*

	Present study (*N* = 46) (*Argyrosomus regius*)	Ktari, 1970 (*N* = 70) (*Umbrina cirrosa*)	Sonsino, 1891 (*N* = 3) (*Umbrina cirrosa*)
Number of clamps	Between 94/96 and 132/137	Between 100/- and -/130^a^	Ca. 100 pairs
Body length	9404 ± 1631 (6169–13 801)	(6000–10 000)	(10 000–12 000)
Body width	700 ± 120 (388–888)	(800–1500)	–
Haptor length	4083 ± 750 (2861–6071)	(2500–4000)	–
Posteriormost clamp length	42 ± 3 (37–48)	60	–
Posteriormost clamp width	57 ± 3 (51–62)	60	–
Testes length	90 ± 11 (64–110)	95	–
Testes width	59 ± 8 (42–79)	75	–
Number of testes	52 ± 7 (38–71)	(60–70)	–
Germarium length	1169 ± 225 (609–1604)	–	–
Germarium width	295 ± 49 (177–387)	–	–
Egg length	180 ± 7 (166–198)*	(180–200)	–
Egg width	69 ± 6 (60–87)*	(75–90)	–

Data expressed as mean ± s.d. (range).

*Intrauterine eggs.

a Haptor with 100–130 clamps in each side with a minimum difference from 4 to 8 between both sides (Ktari, 1970).

Post-larval development of *S. pancerii* is characterized by: progressive acquisition of haptoral clamps combined with increases in body size, expansion and bifurcation of the gut, loss of the larval haptor, protandrous development of the genitalia and finally formation of the vitellaria. This developmental sequence is generally consistent with previous studies on microcotylids (Sproston, 1946; Thoney, 1986; Thoney and Munroe, 1987; Ogawa, 1988; Roubal and Diggles, 1993; Repullés-Albelda *et al*., 2011). Nevertheless, some developmental events, such as terminal lappet loss or parasite maturity, occurred in older stages than in other microcotylids. The terminal lappet is generally lost at 2–13 CPN stages in other microcotylids (Thoney, 1986) whereas it was retained up to 17 CPN stages in *S. pancerii.* These differences are likely related to the comparatively higher clamp pair number reached by *S. pancerii* at the end of development (i.e. 130–132/137 clamps per haptor side; Ktari, 1970; present study). In fact, the terminal lappet loss in *S. pancerii* occurs when about 12% of the maximum clamp pair number has been developed, which fits with previous records on other microcotylids (between 10 and 20% of the maximum clamp pair number; Remley, 1942; Thoney, 1986; Thoney and Munroe, 1987; Roubal and Diggles, 1993; Repullés-Albelda *et al*., 2011). By contrast, parasite maturity is achieved at comparatively older stages in *S. pancerii* than in most microcotylid species, even when the proportion between CPN at maturity and the maximum CPN is considered. While parasite maturity generally occurred between 15 and 45 CPN stages, when 50–70% of the clamps are developed (Repullés-Albelda *et al*., 2011), *S. pancerii* reach maturity at 94/96 CPN stages with at least 70% of the maximum CPN (Ktari, 1970; present study). Despite fitting within the general range of microcotylids, *S. pancerii* is one of the latest to reach maturity, only coinciding with *Polylabroides multispinosus* Roubal, 1981 (Roubal and Diggles, 1993) and *Microcotyle hiatulae* Goto, 1894 (Thoney and Munroe, 1987) which, however, are characterized by a smaller size and a lower clamp pair number.

Developmental data have been reported for 8 out of the 218 microcotylid species listed in the World Register of Marine Species (WoRMS Editorial Board, 2022), but only one of them, *Diplostamenides spinicirrus* (MacCallum, 1918), infects a sciaenid host. The current study describes the development of the unique asymmetric microcotylid reported to date. The combination of morphological features in *S. pancerii*, i.e. haptoral asymmetry, large body size and a high number of clamps is rare among microcotylids (Yamaguti, 1963). Indeed, only 3 of the microcotylids with known development compare well for at least one of the exclusive features (i.e. body length, clamp pair number or the maximum length/CPN ratio). Regarding body size, *S. pancerii* coincides in length with *D. spinicirrus*, a parasite of the freshwater sciaenid *Aplodinotus grunniens* (Rafinesque, 1819), but the maximum CPN of the latest (99 pairs of clamps; Remley, 1942) is substantially lower. According to the ratio between maximum length and CPN, increments of body length per CPN in *S. pancerii* (ratio = 80–100 for the long haptor side) can partially overlap those of *Bivagina tai* (Yamaguti, 1938) (ratio = 61–80, max. length = 4000 *μ*m and max. CPN = 50–65; Ogawa, 1988) and *Microcotyle sebastis* Goto, 1894 (ratio = 92, max. length = 3300 *μ*m and max. CPN = 36; Thoney, 1986), although both have a lower length and CPN. The large CPN of *S. pancerii* stands out as exceptional among microcotylids overall because only 2 other species within this family bear more than 100 pairs of clamps; *Cynoscionicola heteracanta* (Manter, 1938) and *Cynoscionicola longicauda* (Goto, 1899), both from sciaenid fish (based on the morphological descriptions from Yamaguti, 1963). The high clamp number of these microcotylids from sciaenid fish suggests that host traits affect their attachment strategy. Morphological features of the gills of the sciaenids may lead to similar adaptations such as the increase in the number of clamps which may occur relatively fast, based on the high number of developing clamps (between 2 and 5 clamps) observed throughout post-larval development.

The haptor of monogeneans determines the attachment to their hosts and their behaviour (Kearn, 2004). In *S. pancerii*, the haptor is characterized by a high number of clamps, but this species also exhibits a slight haptoral asymmetry in the arrangement and size of the clamps, which is one of the key differential features of *Sciaenacotyle* spp. within the Microcotylidae (Mamaev, 1989). The asymmetry in the clamp arrangement is a common diagnosis feature of other mazocraeid families as the heteraxinids (Yamaguti, 1963), which also show differences in the clamp size at both haptor sides. Detailed analysis of the asymmetry during the development of microcotylid and heteraxinid species, however, reveals relevant differences related to the first register of the asymmetry. The asymmetrical haptor of *S. pancerii* was mainly observed from mature stages, while the haptor of most heteraxinids is asymmetrical from the early developmental stages (Ogawa and Egusa, 1981; Thoney, 1988). Asymmetrical monogeneans tend to minimize dislodgment risks by attaching the largest clamp row upstream (Kearn, 2004). The morphology of the haptor is, therefore, likely to condition the attachment of these parasites to their hosts, so its variability throughout development may involve a change in the attachment strategies. The symmetrical clamp arrangement in the haptor of *S. pancerii* at early developmental stages (<39 CPN) suggests that the haptoral asymmetry is not so decisive for parasite attachment in these life-history stages and the clamp addition, as well as the increases in the clamp width, is probably enough to strengthen the attachment to the host gills. The asymmetrical arrangement and size of the clamps in mature stages may, thereafter, allow the parasite to optimize the attachment when it becomes larger. *Sciaenacotyle pancerii* seems therefore to exhibit a mixed attachment strategy between microcotylids and heteraxinids, combining the high number of clamps (typical of microcotylids) with the asymmetry in the clamp size and arrangement (common in heteraxinids). This mixed strategy may result from the parasite adaptation to a large and fast-growing host and is supported by the phylogenetic closeness of Microcotylidae and Heteraxinidae (Olson and Littlewood, 2002) and the isolated position of the genus *Sciaenacotyle* [represented by *S. sciaenicola* (Murray, 1932)] within most microcotylids (see Hayward *et al*., 2007). The haptoral asymmetry is, therefore relevant for discriminating among this and other microcotylids while not so useful to distinguish *Sciaenacotyle* among mazocraeids. The asymmetry features (i.e. differences in number, size and arrangement of clamps) and the developmental pattern of asymmetry may, however, be more use useful in the field. These features, together with the exclusive number of clamps can be more complete and informative for discriminating between mazocraeids.

## Data Availability

Data available on request from the authors.
